# Multiple Ulcers Associated With Deep Vein Thrombosis Secondary to Herpes Zoster Infection of the Lower Limb

**DOI:** 10.1002/ccr3.70264

**Published:** 2025-05-01

**Authors:** Makoto Kondo, Koji Habe, Keiichi Yamanaka

**Affiliations:** ^1^ Department of Dermatology Mie University Graduate School of Medicine Tsu Mie Japan

**Keywords:** deep vein thrombosis, herpes zoster, lower limb, rheumatoid arthritis

## Abstract

Persistent limb swelling and ulceration following herpes zoster may indicate the development of deep vein thrombosis, highlighting the importance of early diagnosis and initiation of anticoagulant therapy. Pain and reduced mobility are significant contributors to thrombosis, underscoring effective pain management's critical role in the prevention of deep vein thrombosis.

## 1 Case Presentation

**FIGURE 1:**
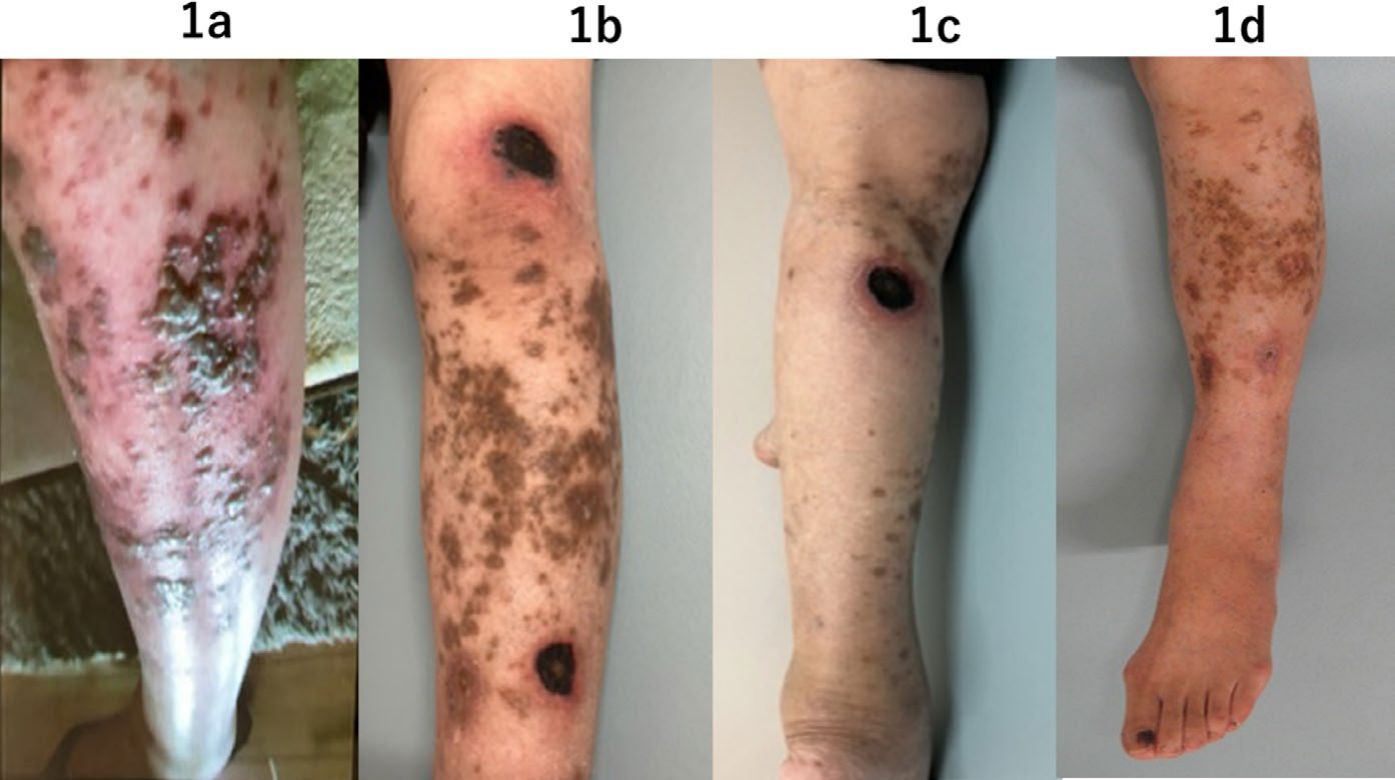
(a) This image shows the left lower limb at the onset of herpes zoster. Multiple blisters and blood blisters have formed on the outer side of the limb, accompanied by redness and swelling. (b) This is the initial finding at our hospital, 4 months after the onset of herpes zoster. The left lower limb exhibits swelling, band‐like pigmentation, and ulcers with necrosis and crusting in a circular pattern. (c) In the left popliteal fossa, a large necrotic crust is visible. Ultrasound revealed a massive thrombus just beneath this area. (d) This image shows the left lower limb after complete epithelialization. The edema has subsided, but significant hyperpigmentation remains.

**FIGURE 2:**
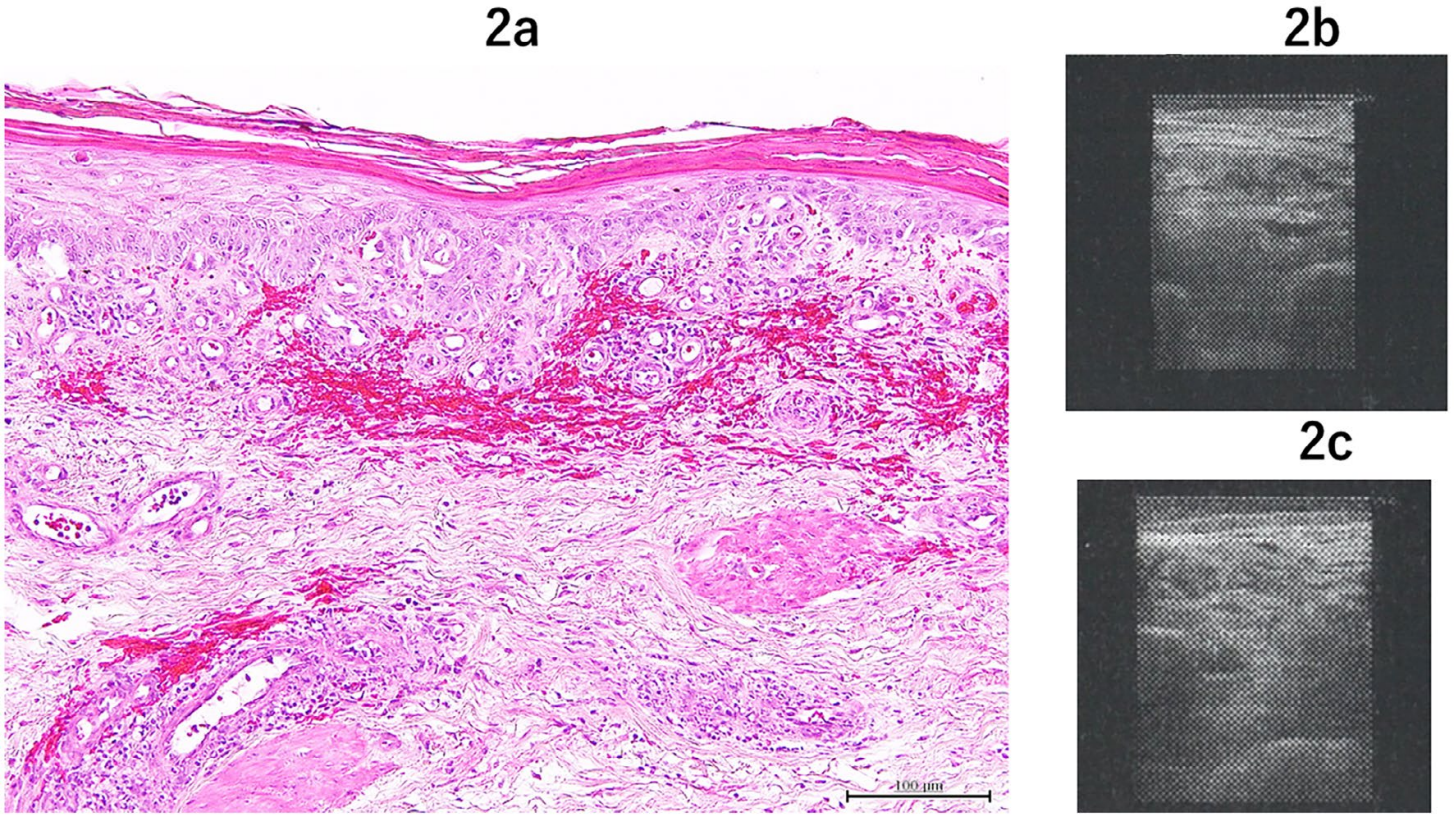
(a) In the dermis, infiltration of inflammatory cells around blood vessels, extravasation of red blood cells, and nuclear dust are observed. Additionally, collagen fiber degeneration is present. (b) An image showing multiple thrombi filling the blood vessels. (c) A large saccular thrombus filling the vessel.

A 76‐year‐old woman with a history of rheumatoid arthritis (RA) presented with painful multiple blisters on her left lower limb (Figure 1a). Her RA had been well‐controlled with methotrexate and prednisolone. The patient was diagnosed with herpes zoster (HZ) and completed a 7‐day course of Amenamevir. Although the blisters resolved, multiple painful round ulcers developed 2 months later. Despite treatment with topical silver sulfadiazine, her condition progressively worsened, accompanied by the emergence of depressive tendencies. The pain intensified, causing her to frequently withdraw from daily activities. She was referred to our hospital 4 months after the onset of HZ. Examination of the left lower limb revealed multiple ulcers with black eschar and swelling (Figure 1b,c). Blood tests showed mildly elevated inflammatory markers and normal coagulation factors, with no evidence of active RA (Table 1). A skin biopsy taken from the edge of an ulcer demonstrated inflammatory cell infiltration and collagen fiber degeneration, with no findings indicative of vasculitis or lymphoma (Figure 2a). Ultrasound of the lower limb revealed extensive thrombus formation from the central to the peripheral femoral vein, resulting in complete occlusion (Figure 2b). A 14‐mm granular thrombus was identified in the popliteal vein (Figure 2c), and additional thrombi were observed in the fibular, posterior tibial, and soleal veins. No evidence of atherosclerosis was detected in the blood vessels. Treatment with rivaroxaban was initiated to dissolve the thrombus. The ulcers were managed through mechanical debridement using a curette to remove necrotic tissue, followed by the topical application of silver sulfadiazine to promote necrotic tissue dissolution. Once granulation tissue developed, transferrin spray was introduced to facilitate epithelialization. The ulcers gradually decreased in size, achieving complete epithelialization of all lesions 1 year after referral (Figure 1d). Follow‐up ultrasound revealed a residual thrombus in the left popliteal vein accompanied by collateral circulation, with no thrombi detected in the other veins.

**TABLE 1:** IgM, IgG, CH50, and MMP‐3 are within the normal range, and the elevated CRP and ESR are considered to be due to the ulcer rather than the activity of rheumatoid arthritis.

Test items	WBC	CRP	ANA	RF	Anti‐DNA	IgM	IgG	CH50	MMP‐3	ESR
Test results	2000	4.54	±	85	< 2	39	1051	46.2	44.2	32
Normal range	3300–8600	0–0.14	−	0–15	< 6	46–260	870–1700	31.7–57.6	17.3–59.7	3–15.0
Units	/μL	mg/dL	±	IU/mL	IU/mL	mg/dL	mg/dL	U/mL	ng/mL	mm/h

Abbreviations: ANA, antinuclear antibody; Anti‐DNA, anti‐DNA antibody; CH50, total hemolytic complement activity; CRP, C‐reactive protein; ESR, erythrocyte sedimentation rate; IgG, immunoglobulin G; IgM, immunoglobulin M; MMP‐3, matrix metalloproteinase‐3; RF, rheumatoid factor; WBC, white blood cell CRP.

Reported thrombosis associated with HZ, including cerebrovascular events and deep vein thrombosis (DVT), are rare [1, 2]. Evidence suggests that varicella‐zoster virus may contribute to thrombus formation by causing vascular endothelial damage. Additionally, RA is a well‐recognized risk factor for DVT [3]. RA promotes a prothrombotic tendency through coagulation activation and chronic inflammation. Additionally, RA is associated with an increased risk of atherosclerosis. However, in this patient, blood tests indicated well‐controlled RA activity and histopathological findings showed no evidence of vasculitis. Furthermore, ultrasound findings showed no signs of atherosclerosis. In this case, the involvement of RA in the development of DVT is considered minimal. Instead, vasculitis or hypercoagulability associated with HZ might have initially played a significant role in the DVT formation. The primary trigger for thrombosis in this case was likely reduced physical activity and mobility limitations caused by pain and depressive symptoms. Therefore, effective pain management, particularly for the lower extremities, is critically important. This case highlights a rare instance of extensive DVT resulting from the interplay of multiple risk factors. This underscores the importance of early initiation of anticoagulant therapy in patients presenting with persistent limb swelling and ulceration following HZ.

## Author Contributions

**Makoto Kondo:** conceptualization, data curation, writing – original draft. **Koji Habe:** writing – review and editing. **Keiichi Yamanaka:** project administration, writing – review and editing.

## Consent

Written informed consent was obtained from the patient to publish this report in accordance with the journal's patient consent policy.

## Conflicts of Interest

The authors declare no conflicts of interest.

## Data Availability

The data that support the findings of this study are available from the corresponding author upon reasonable request.
